# Spasmodic Neuralgia of the Face

**Published:** 1895-08

**Authors:** 


### Spasmodic Neuralgia of fhe Face.

M. Josias, in speaking at the Societe de Therapeutique, related the case of a woman, aged sixty, who suffered for eight years from spasmodic neuralgia of the left side of the face. At times the pain was exceedingly acute. The branches of the nerve were sensitive to pressure, while the edge of the gum on the same side was so painful to touch that mastication was rendered impossible; the patient had from an early age suffered from her teeth, so that nearly all had been extracted. To obtain ease she had exhausted nearly all the hypnotics known, and when she had arrived at the hospital, maximum doses of morphia or sulfonal only gave her a few minutes of relief. Remembering that his colleague, M. Jarre, had reported eighteen cures of obstinate neuralgia by resection of the mucous membrane, periosteum, and a portion of the osseous tissue, where the gum was most sensitive, the speaker requested him to take in hand this patient. M. Jarre operated as usual, and the result was immediate; on the same day all pain had disappeared, never to return.—*Paris Cor. Med. Press and Circular.*

